# Quantitative proteomics analysis of young and elderly skin with DIA mass spectrometry reveals new skin aging-related proteins

**DOI:** 10.18632/aging.103461

**Published:** 2020-06-29

**Authors:** Jing Ma, Mengting Liu, Yaochi Wang, Cong Xin, Hui Zhang, Shirui Chen, Xiaodong Zheng, Xuejun Zhang, Fengli Xiao, Sen Yang

**Affiliations:** 1 Department of Dermatology of First Affiliated Hospital, and Institute of Dermatology, Anhui Medical University, Hefei, Anhui, China; 2 Key Laboratory of Dermatology, Anhui Medical University, Ministry of Education, Hefei, Anhui, China; 3 The Center for Scientific Research of Anhui Medical University, Hefei, Anhui, China

**Keywords:** aging, epidermal proteins, skin rejuvenation and aging, proteome, mass spectrometer

## Abstract

Skin aging is a specific manifestation of the physiological aging process that occurs in virtually all organisms. In this study, we used data independent acquisition mass spectrometry to perform a comparative analysis of protein expression in volar forearm skin samples from of 20 healthy young and elderly Chinese individuals. Our quantitative proteomic analysis identified a total of 95 differentially expressed proteins (DEPs) in aged skin compared to young skin. Enrichment analyses of these DEPs (57 upregulated and 38 downregulated proteins) based on the GO, KEGG, and KOG databases revealed functional clusters associated with immunity and inflammation, oxidative stress, biosynthesis and metabolism, proteases, cell proliferation, cell differentiation, and apoptosis. We also found that GAPDH, which was downregulated in aged skin samples, was the top hub gene in a protein-protein interaction network analysis. Some of the DEPs identified herein had been previously correlated with aging of the skin and other organs, while others may represent novel age-related entities. Our non-invasive proteomics analysis of human epidermal proteins may guide future research on skin aging to help develop treatments for age-related skin conditions and rejuvenation.

## INTRODUCTION

Aging is a normal physiological phenomenon related to progressive deficits in various physiological variables such as cellular redox status, immunity, and metabolism, which contribute to disruption of tissue homeostasis. Skin aging is a specific manifestation of organ aging in the human body and results also from the combined effects of the above factors. Specific features of skin aging include thinning of the epidermis, degeneration of elastic tissues, reduction of melanocyte numbers, and impaired barrier function, which manifest as wrinkles, decreased elasticity, dryness, and dyschromia [1]. Many intrinsic and extrinsic factors contribute to skin aging. They include changes in reactive oxygen species (ROS) generation and compensatory antioxidant mechanisms, dysregulated autophagy, chronic inflammation, cell metabolic disorders, and endocrine decline, all of which are impacted by each individual’s genetic makeup and lifestyle habits. On the other hand, environmental factors such as light damage, especially ultraviolet (UV) light exposure, are also major contributors to skin aging.

Cellular senescence as the basis of endogenous (i.e. intrinsic or chronological) aging is largely determined by gradual, age-dependent shortening of the telomeres, small DNA sequences present at the ends of chromosomes. Telomerase enzymes act as reverse transcriptases to extend the telomeres and slow cellular aging; however, this topic is still controversial. For instance, increased telomere length may be associated with increased risk of melanoma, while shortened telomeres may confer greater risk for cutaneous squamous cell carcinomas [2].

ROS are natural byproducts of cellular respiration. Imbalances between ROS generation and elimination can cause DNA mutations and cell damage, hinder protein synthesis, and induce apoptosis of skin cells [3]. Research also showed that ROS can inhibit the production of collagen in aging skin by activating the MAPK-AP-1/NF-κB-TNF-α/IL-6 pathway [4]. In turn, NF-kB activation through the mTORC2/Akt/IKKa pathway was also shown to influence skin aging [5].

Environmental factors can also reduce skin elasticity and increase collagen fiber damage. UV radiation is the main cause of skin photoaging. Interestingly, repeated UV radiation causes damage to the dermis and dermal extracellular matrix by promoting chronic inflammation [6]. UV rays induce oxidative stress in epidermal cells, which leads to peroxidation of membrane lipids. The damaged cells are recognized by the complement system and cause inflammation, leading to macrophage infiltration and activation. Activated macrophages remove damaged cells and release MMPs to degrade the extracellular matrix. UV light can also induce epidermal keratinocytes to release inflammatory cytokines such as IL-1β and TNF-α; accordingly, global gene expression profiling studies have linked photoaging to differential expression of several inflammation-related genes [7, 8].

Through LC-MS-based proteomics and bioinformatics analyses, the present study evaluated differences in the expression of epidermal proteins between healthy young and elderly subjects to identify differentially expressed proteins (DEPs) possibly involved in skin aging. We also addressed the potential implications of our findings on skin aging mechanisms such as oxidative stress, metabolic reprogramming, and chronic inflammation induced by either physiological aging or photoaging.

## RESULTS

### Quantitative protein detection

**Figure 1:**
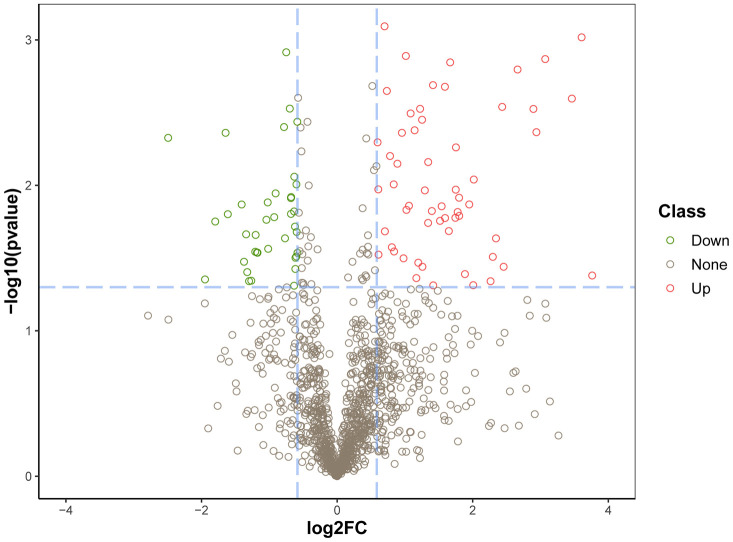
**Identification of DEPs in aged skin samples.** The X axis represents protein difference (log2-transformed fold changes), and the Y axis the corresponding -log10-transformed P values. Red dots indicate significantly upregulated proteins, green dots denote significantly downregulated proteins, and gray dots symbolize proteins with no significant change.

**Figure 2:**
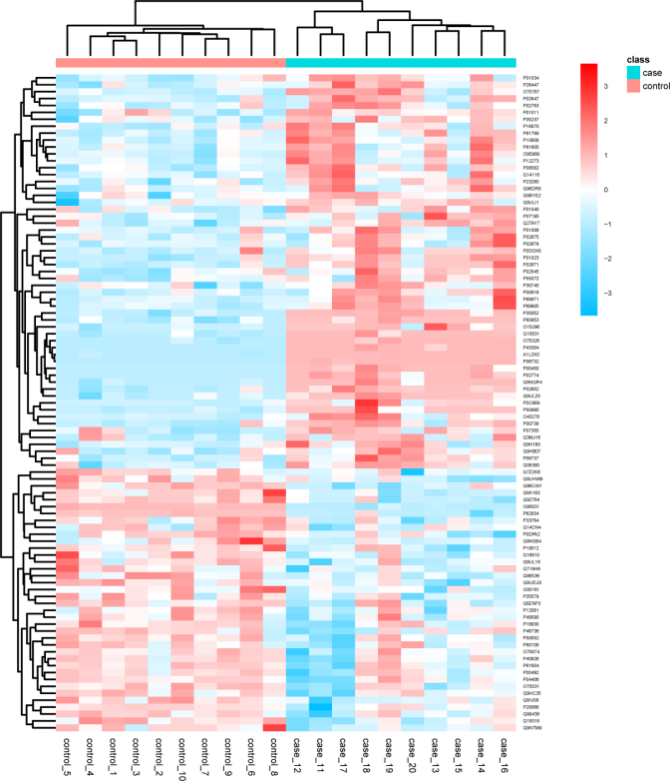
**Cluster analysis chart of the identified DEPs.** Higher red and blue intensities indicate higher degree of upregulation and downregulation, respectively.

Conventional data dependent acquisition (DDA) mass spectrometry was used to establish and analyze a spectral library of human volar skin proteins obtained from 20 healthy subjects, i.e. 10 young and 10 elderly Chinese individuals. As a result, 9005 peptides and 1631 proteins were identified. Supplementary Table 1 lists the details of the proteins produced by DDA. Next, we adopted the data independent acquisition (DIA) method for mass spectrometry data collection. The details of the proteins identified and quantified by DIA are shown in Supplementary Table 2. After calculating the fold difference and P value through the MSstats package, 1270 proteins were further identified (Supplementary Table 3). Fold change ≥ 1.5 and P <0.05 were used as the screening criteria for significantly differentially expressed proteins (DEPs). Compared with the young (control) group, a total of 95 proteins were differentially expressed in the elderly (experimental) group. Among these DEPs, 57 were upregulated and 38 were downregulated (Figure 1). These DEPs were used to draw a cluster analysis chart (Figure 2), which intuitively reflected expression differences between the two groups. Principal component analysis (PCA) showed that the DEPs segregated into two separate clusters that can distinguish the young group from the elderly group (Supplementary Figure 1). Functional categorization and UniProt information for the upregulated and downregulated DEPs are provided in Tables 1 and 2, respectively.

**Table 1:** List of up-regulated proteins.

**Category**	**Accession number**	**Description**	**Gene name**	**pvalue**
Immune and inflammatory response protein	P02763	Alpha-1-acid glycoprotein 1	ORM1	0.020624
	Q08380	Galectin-3-binding protein	LGALS3BP	0.000807
	P01023	Alpha-2-Macroglobulin, Alpha-2-M	A2M	0.031029
	P61769	Beta-2-microglobulin	B2M	0.0021
	P81605	Dermcidin	DCD	0.012168
	P0DOX6	Immunoglobulin mu heavy chain	N/A	0.03629
Blood particles	P69905	Hemoglobin subunit alpha	HBA1	0.00096
	P68871	Hemoglobin subunit beta	HBB	0.002533
	P00738	Haptoglobin	HP	0.002984
	P01008	Antithrombin-III	SERPINC1	0.036303
	P02679	Fibrinogen gamma chain	FGG	0.040763
	P02675	Fibrinogen beta chain	FGB	0.048723
	P02671	Fibrinogen alpha chain	FGA	0.004311
Lipid metabolism related protein	P02652	Apolipoprotein A-II	APOA2	0.001355
	P02647	Apolipoprotein A-I	APOA1	0.002888
	A1L3X0	Elongation of very long chain fatty acids protein 7	ELOVL7	0.006917
	O15296	Arachidonate 15-lipoxygenase B	ALOX15B	0.016722
	P0C869	Cytosolic phospholipase A2 beta	PLA2G4B	0.034032
	Q3MJ16	Cytosolic phospholipase A2 epsilon	PLA2G4E	0.028501
Protease inhibitor	P01011	Alpha-1-antichymotrypsin	SERPINA3	0.004355
	P01040	Cystatin-A	CSTA	0.006286
	P01034	Cystatin-C	CST3	0.007101
	P30740	Leukocyte elastase inhibitor	SERPINB1	0.013816
	P35237	Serpin B6	SERPINB6	0.002979
	O43278	Kunitz-type protease inhibitor 1	SPINT1	0.018128
Calbindin	Q14118	Dystroglycan	DAG1	0.014746
	P26447	Protein S100-A4	S100A4	0.002244
Metal ion related protein	P08582	Melanotransferrin	MELTF	0.01071
	P00450	Ceruloplasmin	CP	0.029955
Signal transduction related protein	O75326	Semaphorin-7A	SEMA7A	0.002046
	P60953	Cell division control protein 42 homolog	CDC42	0.003204
	Q96DR8	Mucin-like protein 1	MUCL1	0.048572
Protease	P16870	Carboxypeptidase E	CPE	0.01083
	P43304	Glycerol-3-phosphate dehydrogenase, mitochondrial	GPD2	0.04563
	P06732	Creatine kinase M-type	CKM	0.041678
	P07195	L-lactate dehydrogenase B chain	LDHB	0.01678
	P06737	Glycogen phosphorylase, liver form	PYGL	0.020716
	P00918	Carbonic anhydrase 2	CA2	0.005483
	P23280	Carbonic anhydrase 6	CA6	0.003541
	Q9NQR4	Omega-amidase NIT2	NIT2	0.015016
	P55072	Transitional endoplasmic reticulum ATPase	VCP	0.01621
Chaperone	P10909	Clusterin	CLU	0.001427
	P50990	T-complex protein 1 subunit theta	CCT8	0.04342
Apoptosis, proliferation and differentiation proteins	Q15631	Translin	TSN	0.009122
	P35052	Glypican-1	GPC1	0.015251
	P02545	Prelamin-A/C	LMNA	0.017547
	P12273	Prolactin-inducible protein	PIP	0.013534
	Q9H190	Syntenin-2	SDCBP2	0.026649
	P07355	Annexin A2	ANXA2	0.005062
Others	P02774	Vitamin D-binding protein	GC	0.023154
	Q9BYE2	Transmembrane protease serine 13	TMPRSS13	0.031752
	Q9UL25	Ras-related protein Rab-21	RAB21	0.009851
	Q2TAY7	WD40 repeat-containing protein SMU1	SMU1	0.004182
	O95969	Secretoglobin family 1D member 2	SCGB1D2	0.001598
	Q9ULI1	NACHT and WD repeat domain-containing protein 2	NWD2	0.010662
	O75787	Renin receptor	ATP6AP2	0.00129
	Q9HB07	UPF0160 protein MYG1, mitochondrial	C12orf10	0.013933

**Table 2:** List of down-regulated proteins.

**Category**	**Accession number**	**Description**	**Gene name**	**pvalue**
Keratin	P19012	Keratin, type I cytoskeletal 15	KRT15	0.00985
	Q6A163	Keratin, type I cytoskeletal 39	KRT39	0.02171
	Q14CN4	Keratin, type II cytoskeletal 72	KRT72	0.04435
	Q92764	Keratin, type I cuticular Ha5	KRT35	0.00436
	Q9NSB4	Keratin, type II cuticular Hb2	KRT82	0.02896
Vesicle transport related protein	O00161	Synaptosomal-associated protein 23	SNAP23	0.01512
	Q99536	Synaptic vesicle membrane protein VAT-1 homolog	VAT1	0.00397
	Q9UEU0	Vesicle transport through interaction with t-SNAREs homolog 1B	VTI1B	0.00366
	Q96CW1	AP-2 complex subunit mu	AP2M1	0.04896
Apoptosis, proliferation and differentiation-related proteins	P62834	Ras-related protein Rap-1A	RAP1A	0.04527
	Q15018	BRISC complex subunit Abraxas 2	ABRAXAS2	0.00122
	O95831	Apoptosis-inducing factor 1, mitochondrial	AIFM1	0.01223
	O75531	Barrier-to-autointegration factor	BANF1	0.00472
Enzymes related to biosynthesis and metabolism	P04406	Glyceraldehyde-3-phosphate dehydrogenase	GAPDH	0.02905
	P00492	Hypoxanthine-guanine phosphoribosyltransferase	HPRT1	0.02192
	P40926	Malate dehydrogenase, mitochondrial	MDH2	0.01773
	O75874	Isocitrate dehydrogenase [NADP] cytoplasmic	IDH1	0.03353
Calbindin	P33764	Protein S100-A3	S100A3	0.01579
Chaperone	P61604	10 kDa heat shock protein, mitochondrial	HSPE1	0.01724
Proteases	Q7Z2K6	Endoplasmic reticulum metallopeptidase 1	ERMP1	0.01922
	P46736	Lys-63-specific deubiquitinase BRCC36	BRCC3	0.0131
	Q6ZNF0	Acid phosphatase type 7	ACP7	0.01572
	P16930	Fumarylacetoacetase	FAH	0.02091
	P80108	Phosphatidylinositol-glycan-specific phospholipase D	GPLD1	0.03758
	P28066	Proteasome subunit alpha type-5	PSMA5	0.02911
	Q99436	Proteasome subunit beta type-7	PSMB7	0.01204
	Q8IV08	5'-3' exonuclease PLD3	PLD3	0.00297
Cytoskeleton related protein	Q9HC35	Echinoderm microtubule-associated protein-like 4	EMAL4	0.01354
	P35579	Myosin-9	MYH9	0.01136
	P50552	Vasodilator-stimulated phosphoprotein	VASP	0.02732
Gene expression related protein	P12081	Histidine—tRNA ligase, cytoplasmic	HARS	0.0395
	P49590	Probable histidine—tRNA ligase, mitochondrial	HARS2	0.04557
	Q9UL18	Protein argonaute-1	AGO1	0.01655
Others	Q16610	Extracellular matrix protein 1	ECM1	0.00873
	P0DPA2	V-set and immunoglobulin domain-containing protein 8	VSIG8	0.02862
	Q9NTM9	Copper homeostasis protein cutC homolog	CUTC	0.03146
	Q719H9	BTB/POZ domain-containing protein KCTD1	KCTD1	0.03087
	Q9UHW9	Solute carrier family 12 member 6	SLC12A6	0.02313

### GO enrichment analysis

**Figure 3:**
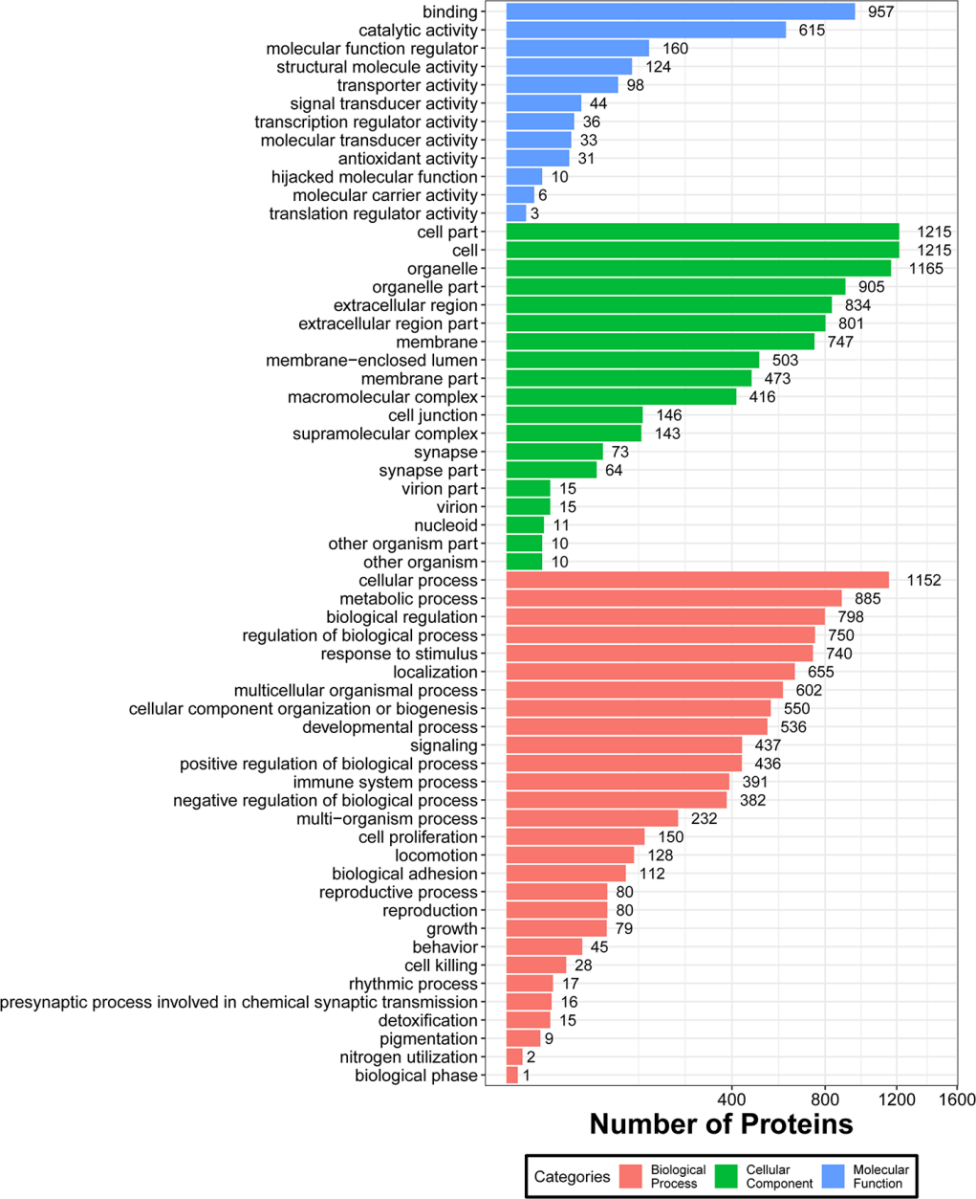
**Functional GO classification of all the identified skin proteins.**

To evaluate the functional significance of all identified proteins, Blast2GO software was used to perform gene ontology (GO) annotation analysis. Protein information and visualization of results are provided in Supplementary Table 4 and Figure 3, respectively. The most enriched biological processes (out of 28 GO terms) were: ‘cellular process’, ‘metabolic process’, ‘biological regulation’, ‘regulation of biological process’, and ‘response to stimulus’. The most enriched cell components (out of 19 GO terms) were: ‘cell’, ‘cell part’, and ‘organelle’. The most enriched molecular functions (out of 12 GO items) were: ‘binding’ and ‘catalytic activity’.

**Figure 4:**
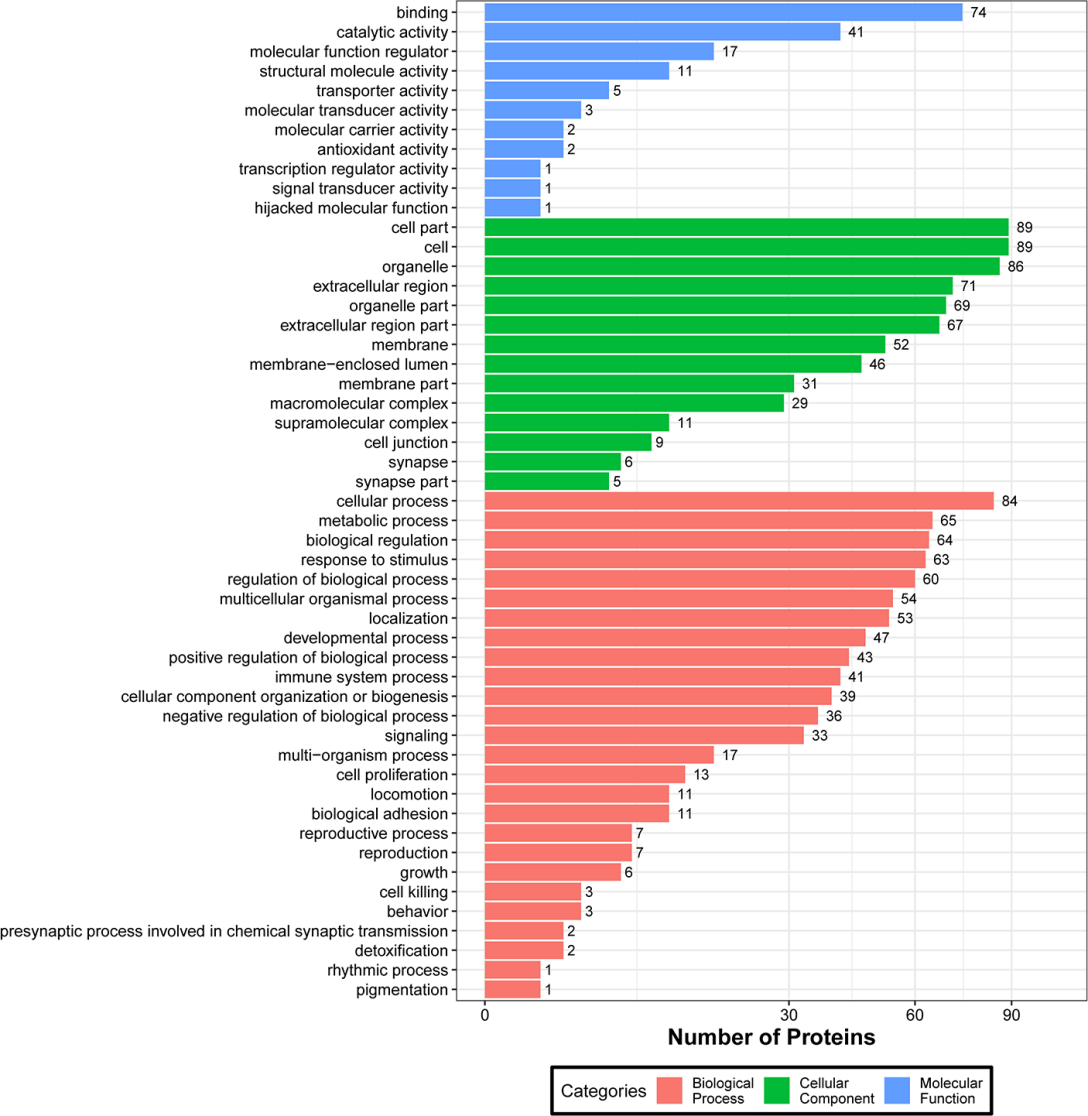
**Functional GO classification of DEPs.**

**Figure 5:**
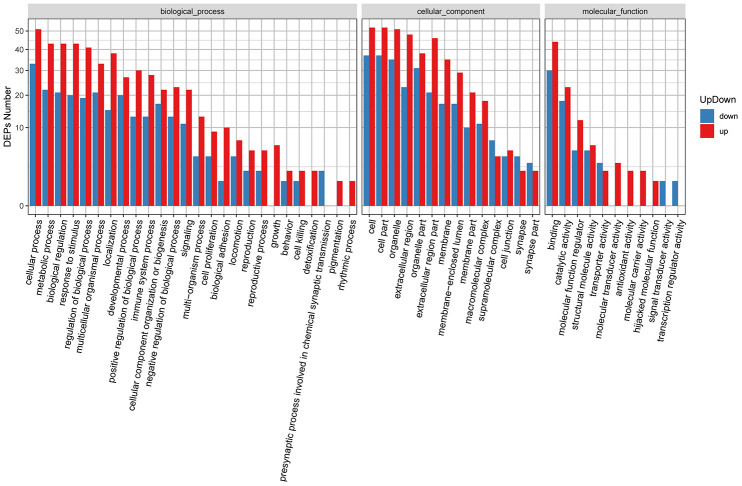
**GO classification of upregulated and downregulated DEPs.**

Next, we carried out GO enrichment analysis of the identified DEPs (Supplementary Table 5). Based on these results, we generated GO functional classification maps to represent all DEPs (Figure 4) and to discriminate up- and down-regulated proteins (Figure 5). It can be seen that in general, both up- and downregulated DEPs intervene in common structural or functional processes. However, unique enrichment by upregulated proteins (e.g. molecular transducer activity, antioxidant activity, molecular carrier activity) or downregulated proteins (e.g. signal transducer activity, transcription regulator activity) was detected for some GO terms within the biological process and molecular function categories (Figure 5).

### KOG classification

**Figure 6:**
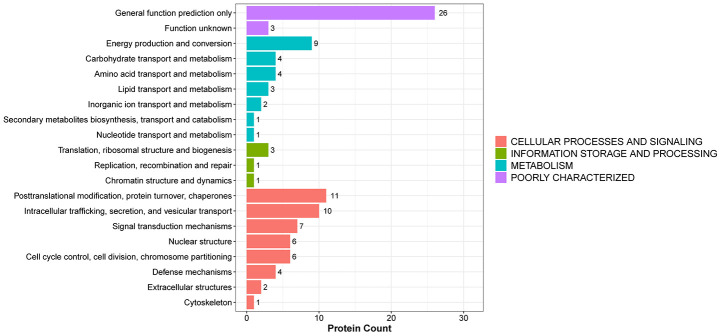
**KOG functional annotation of DEPs.**

Next, we predicted the potential functions of the identified DEPs using EuKaryotic orthologous groups (KOG), a database for the classification of protein orthologs (Supplementary Table 6 and Figure 6). The most represented KOG category was "cell processes and signaling", which showed predominant association of the DEPs with post-translational modification, protein turnover, chaperone activity, intracellular trafficking, secretion, and vesicular transport.

### KEGG pathway analysis

**Figure 7:**
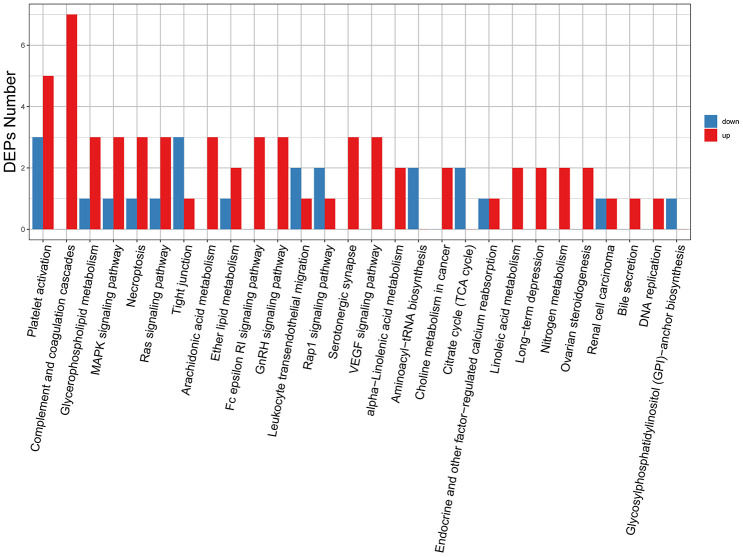
**KEGG pathway classification of DEPs.** The x-axis represents pathway annotation entries, and the y-axis represents the number of DEPs enriched for each pathway term.

**Figure 8:**
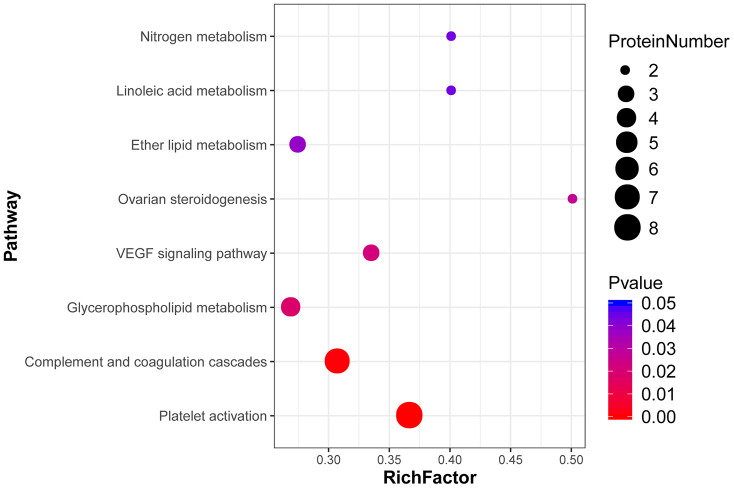
**Top 8 pathways enriched in DEPs from aged skin.** The x-axis indicates the enrichment factor (RichFactor), i.e. the number of DEPs annotated to each pathway divided by all identified proteins annotated to the same pathway. The larger the value, the greater the proportion of DEPs annotated to each pathway. Dot sizes represent the number of DEPs annotated to each pathway.

Then, we conducted KEGG enrichment analysis to further characterize the biological functions of the identified DEPs (Supplementary Table 7). Results showed that upregulated proteins were annotated to 25 major pathways. Among these, the most enriched were ‘complement and coagulation cascade’ and ‘platelet activation’. In turn, downregulated proteins were annotated to 14 major pathways, the most enriched ones being ‘platelet activation’ and ‘tight junction’ (Figure 7). The 8 highest ranked biological functions for our DEP set are shown in Figure 8. Among these pathways, ‘complement and coagulation cascades’ is enriched in SERPINC1, A2M, FGG, FGB, FGA, CLU, PIP, and other proteins whose expression is known to increase with age. On the other hand, several metabolic processes, especially some related to lipid metabolism, were also enriched in various DEPs detected in our aged skin samples.

### Subcellular localization and protein-protein interaction network analyses

**Figure 9:**
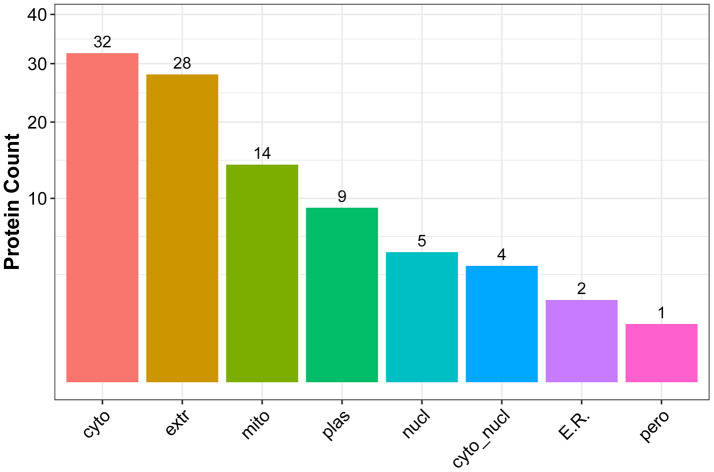
**Subcellular localization of DEPs.** The x-axis represents subcellular structure entries and the y-axis represents the number of DEPs.

We next used WoLF PSORT software to predict the subcellular localization of the identified DEPs (Figure 9). Results showed that the intracellular, extracellular, mitochondria, and plasma membrane compartments were the most represented structures (see Supplementary Table 8 for detailed information).

**Figure 10:**
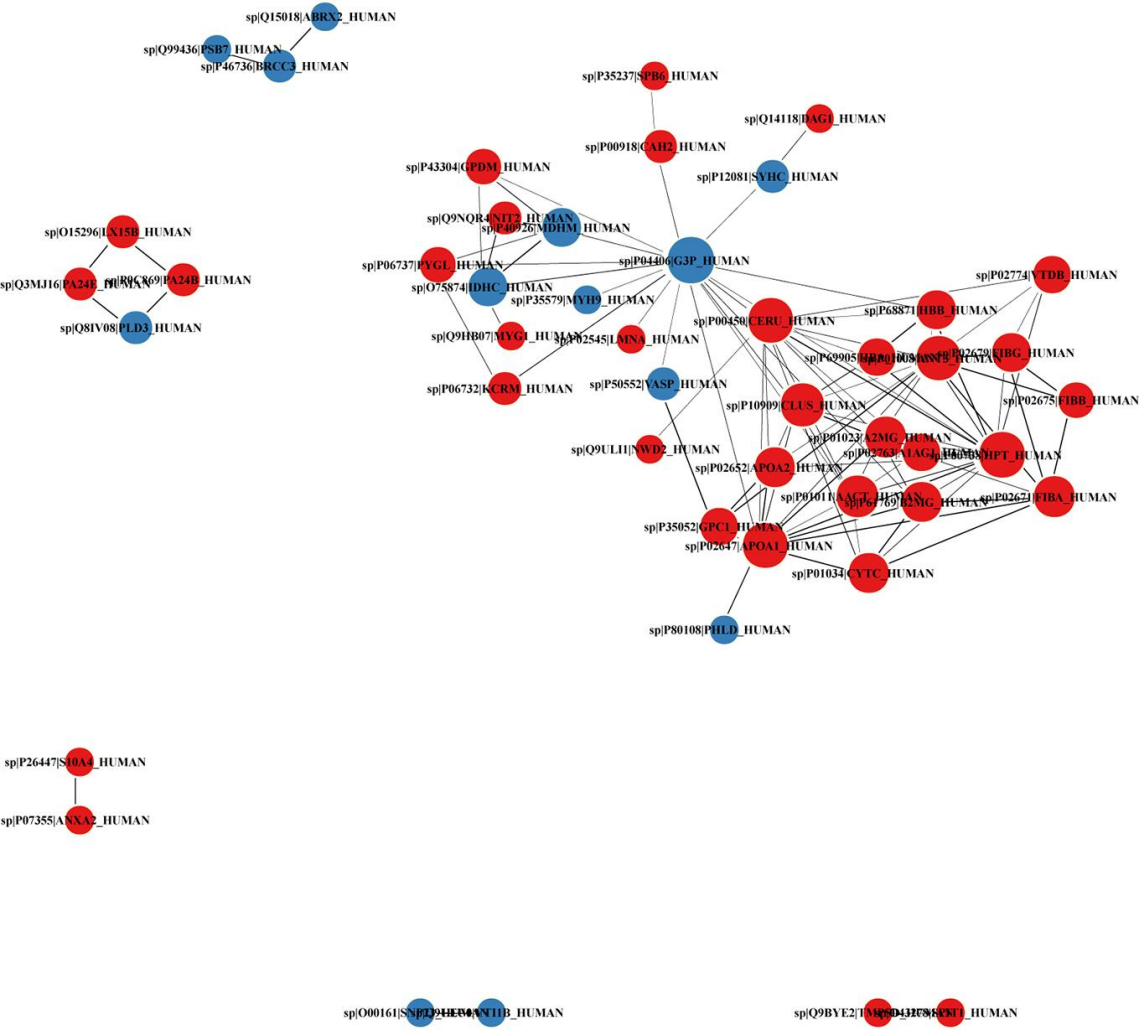
**PPI network diagram of DEPs.** Red and blue nodes indicate upregulated and downregulated proteins, respectively. The size of the circles indicates node degree.

The DEPs were next imported into the STRING database (STRING 11.0) to perform network interaction analysis of protein-protein relationships in the first 100 confidence intervals (Figure 10). Two main clusters were identified, which correspond to two broad functions, namely immunity and inflammation (which included A2M, FGG, FGA, FGB, APOA1, HP, CLU, SERPINC1, B2M), and metabolic processes (which included MDH2, PYGL, CKM, GAPDH, GPD2, IDH1, NIT2). Further analysis on Cytoscape [9] showed that GAPDH, a key enzyme in carbohydrate metabolism, had the highest node degree and represented the top hub protein among all DEPs.

## DISCUSSION

As the largest organ of the human body exposed to the external environment, the skin controls water loss and acts as a physical, anti-microbial, and immune barrier. Over time, the skin is subjected to both natural (chronological) aging and photoaging caused by UV rays. Main features of skin aging are thinning of the epidermis, degeneration of elastic tissue, loss of melanocytes, and decreased barrier function. The molecular basis of skin aging involves cell senescence, oxidative stress, chronic inflammation, reduced DNA repair ability, telomere shortening, point mutations in nuclear mitochondrial DNA, increased frequency of chromosome abnormalities, gene mutations, etc. [10]. In this study, we used LC-MS/MS to compare the expression of skin proteins between young and older people. Results showed that 95 proteins (57 upregulated and 38 downregulated) were differentially expressed in aged human skin.

### Upregulated proteins

Go and KEGG enrichment analyses indicated that several upregulated DEPs identified herein play a role in chronic inflammation, a common contributor to skin aging. KEGG analysis showed that ‘platelet activation’ and ‘complement and coagulation cascade’ were the two most enriched pathways. UV light induces the accumulation of ROS, which are recognized by the complement system, causing chronic inflammation. At the same time, excessive UV induction can directly increase the synthesis of complement factors in the skin [6]. Moreover, ROS production may increase secretion of pro-inflammatory cytokines by aging endothelial cells and vascular smooth muscle cells. This leads to sustained vascular inflammation and stimulates the expression of pro-angiogenic factors like VEGF and PDGF-β [11]. Due to characteristic weakening of the immune function caused by aging, skin homeostasis is compromised and this favors both local and systemic mild chronic inflammation.

We found that several DEPs upregulated in aged skin are related to coagulation, immunity, and inflammation. Alpha-1-acid glycoprotein1 (ORM1) is a 41-43-kDa glycoprotein involved in the liver acute phase response to inflammation. ORM1 is also one of the most important plasma binding proteins, involved in the transport of several endogenous ligands related to inflammation. In addition, ORM1 can interact with leptin receptors [12], and its expression is regulated by inflammatory mediators such as IL-1β, TNF-α, IL-6, and IL-6-related cytokines [13]. Galectin-3 binding protein (Gal-3BP) is a multifunctional, secreted glycoprotein mainly involved in the inflammatory response and tumor transformation and progression. It can be induced by interferons (IFN-α, IFN-β, IFN-γ), TNF-α, and dsRNA/dsDNA [14]. Galectin-3 (LGALS3), a β-galactosidase-binding lectin, is known to promoting cardiac fibrosis, a common event during aging [15]. Alpha-2-macroglobulin (α2M), a tetrameric protein also involved in inflammation, can promote the phagocytosis of bacteria, ROS production, and neutrophil chemotaxis and adhesion [16]. It was proposed that α2M mRNA levels may serve as a biomarker for aging in fibroblasts grown in lung tissue cultures [17]. Beta-2-microglobulin (B2M) is a component of the class I histocompatibility complex (MHC) and contributes to host's immune response to tumors [18]. The immunoglobulin mu heavy chain protein forms the large subunit of IgM, involved in maintaining antigen specificity [19]. Dermcidin (DCD) is constitutively secreted in the skin, has antimicrobial properties and contributes to defense mechanisms that prevent fungal colonization [20].

Many blood particles and coagulation cascade proteins were identified as DEPs in aged skin. These include hemoglobin, haptoglobin (HP), antithrombin III (SERPINC1), and fibrinogen. A normal adult hemoglobin tetramer is composed of two alpha chains (HBA) and two beta chains (HBB). Hemoglobin mediates oxygen transport and is also an indicator of skin’s physiological function [21]. HP is crucial for the elimination of free hemoglobin in the blood and exerts anti-inflammatory and immunomodulatory effects in extravascular tissues [22]. Therefore, upregulation of HP expression may represent a physiological indicator of aging. SERPINC1 regulates the blood coagulation cascade by inhibiting thrombin and other serine proteases [23]. Fibrinogen is synthesized in the liver and released into the blood, where it acts as a critical coagulation factor. It consists of three pairs of different polypeptide chains: fibrinogen alpha chain (FGA), fibrinogen beta chain (FGB), and fibrinogen gamma chain (FGG). In addition to its hemostatic function, fibrinogen can also bind many other proteins, including fibronectin, albumin, thrombospondin, vascular pseudohemophilia factor (vWF), fibrin, FGF2, VEGF, and IL-1, and affect cell migration and adhesion and inflammatory responses [24].

At present, the "brick wall" model is most commonly used to describe the skin barrier. Keratinocytes and their internal structural proteins form the "brick" of the skin barrier, and skin lipids serve as the "mud." Together they maintain skin stability and rejuvenation. Interestingly, studies on lipid metabolism and aging reported that the lifespan of experimental animals can be extended by manipulating genes involved in lipid synthesis (e.g. lipase overexpression), and even by fat removal. Indeed, it was suggested that blood lipids may be used as biomarkers of human aging [25]. Research showed that high plasma levels of apolipoprotein E (APOE), a protein involved in lipid transport and metabolism, are associated with increased risk of cardiovascular disease and may serve as a marker of human aging [26]. In our study, APOA1 and APOA2 were found to be upregulated in aged skin samples. Studies have shown that APOA1 levels are significantly reduced in acne patients [27]. Therefore, we speculate that these two proteins may be associated with reduced sebum secretion in the elderly, leading to a dry and desquamating phenotype. Other upregulated DEPs included fatty acid elongase 7 (ELOVL7), which participates in the synthesis and extension of very long-chain fatty acids [28], and arachidonate 15-lipoxygenase B (ALOX15B), a key enzyme in the metabolism of polyunsaturated fatty acids. The latter finding deserves further scrutiny, since downregulation of ALOX15B gene expression in light-damaged skin has been reported previously [29].

Phospholipase A2 group IVB (PLA2G4B) and phospholipase A2 group IVE (PLA2G4E) are members of the calcium-dependent phospholipase A2 family, also associated with lipid metabolism. Their role in the lipid signaling network in the skin may be mediated by binding to the phospholipase A2 receptor (PLA2R1) to influence pro-inflammatory signaling, autoimmunity, apoptosis, and aging. In this regard, Griveau et al. [30] reported that PLA2R1 knockdown delayed senescence in progerin-expressing human fibroblasts, and concluded that PLA2R1 mediates key premature aging phenotypes through a p53/FDPS pathway. In addition, Sukocheva et al. [31] showed that PLA2R1 induces the activation of mitochondrial proteins controlled by Janus kinase 2 (JAK2) and estrogen-related receptor alpha (ERRα), increasing ROS production and promoting aging. Therefore, upregulation of PLA2G4B and PLA2G4E may contribute to skin aging through lipid metabolism pathways and/or lipid signaling networks.

Various protease inhibitors were also differentially upregulated in aged skin samples. Alpha-1-antichymotrypsin (SERPINA3) is present in senile plaques and accelerates the formation of Aβ fibers associated with Alzheimer's disease (AD) [32]. In addition, SERPINA3 was shown to promote migration and invasion of melanoma cells [33]. Leukocyte elastase inhibitor (LEI/SERPINB1) and SERPINB6 also function as intracellular serine protease inhibitors that protect cells against damage induced by proteases released into the cytoplasm in response to cellular stress [34, 35]. Research showed that high SERPIB1 and SERPIB6 expression is associated with higher levels of amyloidosis in women with AD [36]. Cystatins function as reversible cysteine protease inhibitors that regulate cathepsin activity, autophagy, immune function, and elicit protection against neurodegenerative diseases. Studies have shown that mutations in the CSTA gene encoding Cystatin-A are associated with acral peeling skin syndrome and exfoliative ichthyosis [37]. Consistent with our findings, a study showed that cystatin-A content in the facial skin of the elderly was much higher than in younger people [38]. Meanwhile, cystatin-C expression, which was also upregulated in our aged skin samples, has been linked to brain aging in previous studies [39]. Kunitz-type protease inhibitor 1 (SPINT1), a type II transmembrane serine protease inhibitor, has been shown to be involved in the development of several types of cancer, such as squamous cell carcinoma and colorectal cancer. Of note, alterations in the SPINT1 gene are highly prevalent in skin cutaneous melanoma (SKCM) patients and may promote metastatic invasion [40].

Among the metal-binding proteins found to be upregulated in aged skin was melanotransferrin (MELTF) and ceruloplasmin (CP). Melanotransferrin is a transferrin homolog involved in iron metabolism and transport, whose activity impacts angiogenesis, plasminogen activation, cell migration, and tumorigenesis [41]. Ceruloplasmin (CP) acts as a plasma oxidase and can form stable complexes with many proteins [42]. We also detected upregulation of various signal transduction-related proteins in aged skin, with potential roles in carcinogenesis. Semaphorin 7A (SEMA7A) is highly induced via mTOR signaling by the EGFR pathway. Increased mTOR activity is associated with the aging process. Activation of mTOR downregulates lysosomal degradation (autophagy), which weakens the removal of damaged organelles, toxic substances, and pathogens [43] [11]. The SEMA7A-integrin β1 (ITGB1) axis also plays a key role in ERK activation and inhibition of apoptosis [44]. Cell division control protein 42 homolog (CDC42) is a member of a small GTPase family associated with cell cycle control and is dysregulated in a variety of human cancers. It exerts positive regulation of cell proliferation, migration and invasion, angiogenesis, tissue transformation, and tumor growth [45]. The glycoprotein mucin-like protein 1 (MUCL1) is mostly expressed in breast tissue. It stimulates proliferation, has anti-apoptotic actions, is involved in the endoplasmic reticulum stress response, and has prognostic value in breast cancer. Studies have shown that the phosphoinositide 3-kinase/Akt pathway controls MUCL1 expression downstream of HER2. MUCL1 also stimulates focal kinase (FAK) and Jun NH2 terminal kinase (JNK) signaling and c-Jun phosphorylation [46].

ATP6AP2 participates in the assembly of the vacuolar (V)-ATPase proton pump, thereby controlling proteolysis, autophagy, and glycosylation events. Accordingly, missense mutations in ATP6AP2 lead to decreased V-ATPase activity and defects in autophagy and glycosylation [47, 48]. Carboxypeptidase E (CPE) is involved in the biosynthesis of various neuropeptides and hormones in the endocrine and nervous systems, and is associated with hyperinsulinemia and insulinoma [49]. GPD2 (glycerol 3-phosphate dehydrogenase 2) is located in the inner mitochondrial membrane and catalyzes the conversion of cytoplasmic glycerol 3-phosphate (G3P) into dihydroxyacetone phosphate. It is connected to glycolysis, oxidative phosphorylation, and fatty acid metabolism as an intermediate metabolic enzyme for glycerophosphate shuttling. GPD2 can also promote ROS production at the respiratory chain and hence increase oxidative stress [50]. Creatine kinase, M-type (CKM) is a cytoplasmic enzyme that reversibly catalyzes phosphate transfer between ATP and various phosphagens. Accumulation of oxidatively modified (carbonylated) proteins is a hallmark of cellular and tissue aging, and carbonylation of CKM has been reported in previous studies on skeletal muscle aging [51]. Lactate dehydrogenase B (LDHB) was shown to exert transcriptional modulation after cuticle removal in aging skin, suggesting that it may be related to epidermal repair [52]. Glycogen phosphorylase L (PYGL) is associated with degradation of liver glycogen. Studies have shown that hypoxia induces glycogen metabolism with subsequent PYGL upregulation. In turn, PYGL depletion and concomitant glycogen accumulation lead to elevated ROS levels and induction of p53-dependent senescence [53]. Carbonic anhydrase 2 (CA2) and CA6 catalyze reversible hydration of carbon dioxide, a process related to the regulation of skin pH. In addition, altered CA2 expression has been linked to inflammatory skin diseases [54]. Nitrilase family member 2 (NIT2) has ω-amidase activity and possesses inhibiting effects on tumor cell growth [55]. Valosin-containing protein (VCP), a member of the AAA-ATPase family of proteins, plays a key role in ubiquitin-dependent protein degradation, cell cycle, inhibition of apoptosis, and DNA damage response. Skin immunohistochemical studies on patients with amyotrophic lateral sclerosis (ALS) have shown that increased VCP expression correlates with the progression of this neurodegenerative disease [56, 57].

Aged skin samples showed upregulated expression of clusterin (CLU), a chaperone that inhibits both accumulation of elastin in photoaged skin [58] and melanogenesis through MITF/tyrosinase downregulation [59]. These actions may be related to characteristic phenotypes (e.g. reduced elasticity and discoloration) of aging skin. In addition, CLU is associated with lipid transport, apoptosis, tissue remodeling, stress response, inflammatory skin diseases, diabetes, and metabolic syndrome [60]. Chaperonin containing TCP1 subunit 8 (CCT8) is a component of the chaperonin-containing T-complex (TRiC), which regulates telomerase transport and stabilizes telomere extension [61]. Translin (TSN) is a DNA-binding protein that can form multimers with Translin-related factor X (TRAX) and modulates RNA processing and DNA repair. Translin was shown to restrict the proliferation and differentiation of mesenchymal cells [62].

Glypican 1 (GPC1) is associated with control of cell growth, proliferation, and differentiation. A study showed that GPC1 is the predominant GPC isoform in human keratinocytes and its expression decreases significantly with age [63]. This result is contrary to our findings, thus larger sample studies are required. Lamin A/C (LMNA) are structural components of the nuclear lamina and influence chromatin organization and telomere dynamics. Mutations in LMNA can cause diverse pathologies, including Hutchinson-Gilford premature aging syndrome [64]. The function of prolactin induced protein (PIP), another DEP with upregulated expression in aged skin, is not fully clear. PIP is found in a variety of human secretions, has immunoregulatory functions, and is abundantly expressed in human cancers, especially breast cancer, where it exerts tumor-promoting actions [65]. A study showed that PIP can damage the skin barrier and induce keratinocyte proliferation [66]. Annexin A2 (ANXA2) is a member of the annexin family. Its functions include protecting S100A10 from ubiquitination and proteasomal degradation [67]. In addition, studies suggested that ANXA2 affects fibroblast migration and inhibits keloid fibroblast proliferation [68, 69]. Syndecan binding protein 2 (SDCBP2) binds to phosphatidylinositol 4,5-bisphosphate (PIP2) and may play a role in the organization of nuclear PIP2, cell division, and cell survival [70].

Several other functional proteins were also found to be upregulated in aged skin. GC (vitamin D-binding) protein (GC) is involved in the transport and storage of vitamin D, clearance of extracellular actin, and chemotaxis of neutrophils and macrophages during inflammation [71, 72]. Transmembrane serine protease 13 (TMPRSS13) is a membrane-anchored serine protease with roles in skin development and maintenance of barrier function homeostasis [73]. Ras-related protein Rab-21 (RAB21) is involved in the regulation of cell adhesion and migration, and its overexpression can enhance the production of Aβ [74]. Lipophilin B (SCGB1D2), a member of the secretoglobin superfamily, is expressed almost specifically in breast tissue and is upregulated in breast cancer and female genital tract tumors [75]. Dystroglycan (DAG1) is a transmembrane protein that connects the extracellular matrix to the cytoskeleton. It is involved in the assembly and maintenance of the basement membrane structure necessary for tissue morphogenesis and signal transmission across the plasma membrane [76]. The functions of WD40 repeat-containing protein SMU1 (SMU1), NACHT and WD repeat domain-containing protein 2 (NWD2), and UPF0160 protein MYG1, mitochondrial (C12orf10) are unclear. SMU1 functions as a DNA replication regulator and spliceosomal factor and is an essential host protein for influenza virus infection [77].

### Down-regulated proteins

We detected 38 downregulated DEPs in our aged skin samples. Among them were five keratins (KRT15, KRT39, KRT72, KRT35, and KRT82), which is consistent with decreased epidermal barrier function with aging. Besides keratins, actin, proline-rich small protein 1 (SPRR1), S100 proteins, and loricrin are among the best studied skin barrier proteins. Several S100 proteins were downregulated in aged skin. They contain two EF-hand calcium binding motifs and are involved in the regulation of many cellular processes, such as cell cycle progression and differentiation. S100 proteins are one of the components of the cornified envelope (CE) in the skin [78]. Our results found that S100A3 expression decreased with age, whereas S100A4 was upregulated. S100A3 may promote calcium-dependent epidermal cell differentiation, leading to hair shaft and hair cuticular barrier formation [79]. Decreased expression of S100A3 in the aging epidermis also reflects weakening of its barrier function.

Extracellular matrix protein 1 (ECM1) supports the structure and function of human skin. In the epidermis, ECM1 affects the proliferation and differentiation of keratinocytes. In the dermis, ECM1 binds to perlecan, type IV collagen, and laminin 332 and acts as a “biogel” helping to assemble and combine the basement membrane and dermal interstitium [80]. ECM1 participates in various pathological and physiological skin processes, including scar formation and aging. ECM1 is also involved in the excessive angiogenesis and vasodilation observed in psoriasis [81]. Research has shown that ECM1 expression in human skin decreases with age and increases with UV exposure, although the underlying mechanisms and effects remain controversial [82].

Interestingly, all the DEPs with vesicle trafficking functions identified in this study were downregulated with age. These included synaptosomal-associated protein 23 (SNAP-23), synaptic vesicle membrane protein VAT-1 homolog (VAT-1), vesicle transport through interaction with t-SNAREs homolog 1B (VTI1B), and AP-2 complex subunit mu (AP2M1). SNAP-23 regulates the exocytosis of various inflammatory mediators by mast cells [83]. VAT-1 positively regulates calcium-dependent keratinocyte activation during epidermal repair [84]. VTI1B, syntaxin 8, syntaxin 7, and endobrevin/VAMP-8 form a SNARE complex involved in fusion of late endosomes in vitro. Progressive neurodegeneration was observed in the peripheral ganglia of Vti1b-deficient mice [85, 86]. A recent quantitative proteomics analysis study found that AP2M1 is involved in the transmission of secretory signals produced by senescent cells. This property may be related to the cdk4-EZH2-AP2M1 pathway that regulates the mechanism of senescence escape [87].

Other skin DEPs downregulated with age participate in cell proliferation, differentiation, and apoptosis. Rap1A, a member of the Ras oncogene family, is a small G protein involved in cell differentiation. Studies have shown that Rap1A regulates osteoblast differentiation through ERK/p38 signaling and promotes the transformation of epithelium to mesenchyme through the AKT signaling pathway [88, 89]. BRISC complex subunit Abraxas 2 (ABRAXAS2) functions as a central scaffold protein that assembles various components of the BRISC complex and retains it in the cytoplasm. It also regulates BRCA1 localization to damaged DNA sites [90]. Apoptosis-inducing factor, mitochondria-associated 1 (AIFM1) is a NADH oxidoreductase that acts as a pro-apoptotic factor following release from the mitochondria [91]. Barrier-to-autointegration factor 1 (BANF1) is involved in the upregulation of keratinocyte proliferation in psoriatic lesions [92].

Other downregulated DEPs included enzymes related to biosynthesis and metabolism. Among these, glyceraldehyde 3-phosphate dehydrogenase (GAPDH) was the most interconnected (hub) node in our PPI analysis. GAPDH is a key enzyme in anaerobic glycolysis and its activity influences many processes, including carbohydrate metabolism, apoptosis, autophagy, vesicle trafficking, nuclear membrane fusion, signal transduction, transcriptional regulation, and telomere stability and maintenance [93–96]. Dysregulated GAPDH expression has been associated with cancer, neurodegenerative diseases, metabolic syndrome, and inflammation [93, 97, 98]. The increase in GAPDH transcription during hypoxia is mediated by upregulation of hypoxia-inducible factor 1 (HIF-1). The HIF-1 pathway activates genes that promote survival during hypoxia, thereby extending lifespan [99]. Therefore, GAPDH downregulation in aging skin may reflect decreased glycolytic capacity and reduced rejuvenation ability upon activation of the HIF-1 signaling pathway.

Hypoxanthine phosphoribosyltransferase 1 (HPRT1) plays a key role in purine nucleotide production through the purine rescue pathway [100]. Malate dehydrogenase, mitochondrial (MDH2) catalyzes the reversible oxidation of malate to oxaloacetate in the tricarboxylic acid (TCA) cycle to provide energy to the cells [101]. Isocitrate dehydrogenase 1 (IDH1) catalyzes the oxidative decarboxylation of isocitrate to 2-oxoglutaric acid. IDH1 expression dysregulation is observed in many cancer types, and a decrease in IDH1 expression is highly correlated with the pathogenesis of early skin tumors [102].

Heat shock protein family E (Hsp10) Member 1 (HSPE1) has chaperone function and complexes with Hsp60 to assist protein folding in the mitochondrial matrix [103]. Proteasome 20S subunit alpha 5 (PSMA5) and proteasome 20S subunit beta 7 (PSMB7) are components of the 20S proteasome complex, involved in the proteolytic degradation of most intracellular proteins. The 26S proteasome formed when the complex is associated with two 19S regulatory particles can hydrolyze misfolded or damaged proteins [104]. Consistent with our findings, a study reported age-related decrease in proteasome activity in dermal fibroblasts [105].

Other DEPs downregulated in aged skin included copper homeostasis protein cutC homolog (CUTC), a shuttle protein that along with CP helps maintain copper homeostasis and is essential for energy generation, ROS removal, coagulation, and connective tissue cross-linking [106]; endoplasmic reticulum metallopeptidase 1 (ERMP1), related to the unfolded protein response and oxidative stress defense [107], and fumarylacetoacetate hydrolase (FAH), an essential enzyme in metabolic pathways that degrade aromatic compounds [108]. Glycosylphosphatidylinositol-specific phospholipase D1 (GPLD1) hydrolyzes inositol phosphate bonds in proteins anchored by phosphatidylinositol (GPI-anchor), thereby releasing the anchor protein from the membrane. GPI anchoring protein expression is essential for proper skin differentiation and maintenance [109]. Phospholipase D3 (PLD3) is a member of the PLD superfamily, catalyzing the hydrolysis of membrane phospholipids. PLD3 participates in the sorting of endosomal proteins and regulates APP processing [110, 111]. Overexpression of PLD3 leads to a significant reduction of intracellular amyloid β precursors, which can reduce the risk of Alzheimer's disease [112]. Therefore, we consider PLD3 as a negative regulator of the aging process.

Several cytoskeleton-related proteins were also differentially downregulated in aged skin. Echinoderm microtubule-associated protein-like 4 (EMAL4) stabilizes microtubules [113]. Myosin heavy chain 9 (MYH9) promotes cytoskeletal reorganization during cell spreading [114]. Vasodilator-stimulated phosphoprotein (VASP) is a member of the actin regulatory Ena/VASP protein family, which mediates actin filament elongation and regulates fibroblast migration. In addition, VASP is necessary for PKA-mediated platelet aggregation inhibition [115]. Other downregulated DEPs are involved in post-transcriptional regulation of gene expression. Both histidyl-tRNA synthetase (HARS) and HARS2 are members of the aminoacyl tRNA synthetase (ARS) family that attaches specific amino acids to their related tRNA molecules during protein synthesis [116]. Argonaute RISC component 1 (AGO1) binds to miRNAs in the RISC complex to mediate post-transcriptional gene silencing [117].

The functions of other DEPs found to be downregulated in aged skin are less clear. For example, V-set and immunoglobulin domain-containing 8 (VSIG8) may regulate hair follicle keratinization and hair stem differentiation [118, 119]. Potassium channel tetramerization domain-containing 1 (KCTD1) negatively regulates the AP-2 family of transcription factors and promotes adipogenesis [120]. Mice deficient in solute carrier family 12 member 6 (SLC12A6) showed motor dysfunction, peripheral neuropathy, and sensory motor gating defects [121]. Other poorly characterized proteins included acid phosphatase type-7 (ACP7), a metallohydrolase, and BRCA1/BRCA2-containing complex subunit 3 (BRCC3), a component of the BRCC complex with E3 ubiquitin ligase activity.

The results of our differential protein expression analysis in young vs aging skin may allow distinction between "young factors", i.e. proteins involved in angiogenesis, vesicle transport, skin barrier structure, cell proliferation and differentiation, and "aging factors", i.e. proteins related to chronic inflammation, oxidative stress, DNA damage, mitochondrial dysfunction, and signal transduction. Several of the DEPs identified in aged skin are associated to chronic inflammation, an important factor leading to skin aging, while others are related to the development of cancer or AD. Among the 95 DEPs identified, some had already been linked to skin rejuvenation or aging. For other DEPs, age-related expression was proved in other tissues and organs, but had not yet been demonstrated in skin tissue. In turn, and to the best of our knowledge, many other DEPs identified herein had not previously been related to aging. Therefore, this proteomics study provides reference proteins for future research on skin aging and may help develop treatments for age-related skin conditions and rejuvenation.

## MATERIALS AND METHODS

### Study participants

Twenty healthy Chinese volunteers participated in the study. Mean age was 26.4 ± 3.06 years (range 20-35 years) in the youth group (control) and 59.9 ± 6.24 years (range 50-75 years) in the elderly group. Age grouping was not influenced by gender distribution, as determined by Fisher's exact probability method test (P = 0.370). The inclusion criterion was to have well preserved skin integrity at the sampling site. Donors with skin disease, other systemic diseases, or who experienced allergic reactions to the skin sampling tape were excluded from the study. No humectants or other cosmetics were used on the day of the experiment. The study followed the recommendations of the Medical Ethics Committee of Anhui Medical University, and all subjects provided informed consent.

### Reagents, supplies, and equipment

Sodium dodecyl sulfate (SDS), polyacrylamide, SDS-free L3 lysate, ethylenediamine tetraacetic acid (EDTA), dithiothreitol (DTT), Coomassie brilliant blue G-250, 40% ethanol, 10% acetic acid, acrylonitrile (ACN), and trypsin(Hualiishi Scientific, Beijing, China). Empore™ C18 47 mm extraction discs (Model 2215), Pierce C18 tips (10 μl bed), Thermomixer (MS-100), CentriVap vacuum concentrator, and related accessories were obtained from Thermo Fisher Scientific (Shanghai, China). Ultrafiltration membranes (10K MWCO, 1.5 ml, flat sheet) were obtained from Pall Corp. (NY, USA). A Q Exactive HF mass spectrometer and an UltiMate 3000 HPLC system (Thermo Fisher Scientific, San Jose, CA, USA) were used for sample analyses.

### Sample preparation

Stratum corneum samples from the volar forearm skin were collected using 3M medical tape. To this end, the sampling area was gently cleaned with a sterile cotton ball, the tape was applied onto the skin, and uniform pressure was applied intermittently for 1 min before tape removal. Five collections were sequentially performed at the same location. To minimize variability, in all cases the procedure was carried out by the same technician.

### Protein extraction, quality control, enzymolysis

Protein extraction was performed as follows: (1) tape-skin samples were cut into small pieces (0.5 x 0.5 cm) with a sterile blade and deposited inside a glass plate prior to transfer to a 1.5 ml centrifuge tube; (2) Add appropriate amount of lysis buffer without SDS to the sample, add 2mM EDTA, 1XCocktail, and place on ice for 5 minutes. Then, 10 mM DTT were added and samples were left to soak overnight; (3) following centrifugation at 25,000g, 4 °C for 15 min, the supernatants were recovered and treated with 10 mM DTT on a 56 °C bath for 1 h; (4) samples were then treated with 55 mM IAM, incubated at RT in the dark for 45 min, and centrifuged at 25,000g, 4 °C for 15 min to obtain the final protein solution in the resulting supernatant. Protein concentrations were next measured using the Bradford assay [122], and the purity of the extracted proteins was verified by SDS-PAGE on 12% gels followed by Coomassie blue staining. For proteolysis, 100 μg of protein/sample was hydrolyzed over 4 h at 37 °C in 2.5 μg of Trypsin (protein: enzyme ratio = 40: 1). Trypsin was then added again at the same ratio, and enzymolysis continued at 37 ° C for another 8 h. The hydrolyzed peptides were next desalted using a Strata X column and dried under vacuum.

### High pH reverse-phase separation

All samples were mixed equally (10μg/sample), and 200μg sample was diluted with 2 mL buffer A (5% ACN, pH 9.8) and injected onto a Shimadzu LC-20AB liquid phase system. A 5 μm, 4.6 x 250 mm Gemini C18 column was used for liquid phase separation of samples. Gradient elution was applied at a flow rate of 1 mL/min: 5% buffer B (95% ACN, pH 9.8) for 10 min, 5% to 35% buffer B for 40 min, and 35% to 95% buffer B for 1 min. Phase B lasted 3 min and 5% buffer B was equilibrated for 10 min. The elution peak was monitored at a wavelength of 214 nm and one component was collected every minute. The sample was combined with the elution peak of the chromatogram to obtain 10 components, and then freeze-dried.

### Data-dependent and data-independent acquisition (DDA and DIA) analysis

The dried peptide sample was re-dissolved with buffer A (2% ACN, 0.1% FA), centrifuged at 20,000 g for 10 min, and the supernatant was injected. Separation was performed on a Thermo Corporation UltiMate 3000 UHPLC. The sample first entered a trap column for enrichment and desalting. Then the sample was connected in series with a self-packed C18 column (150 μm inner diameter, 1.8 μm column diameter, 25 cm column length). Peptides were separated at a flow rate of 500 nL/min through the following effective gradient: 0-5 min, 5% buffer B (98% ACN, 0.1% FA); 5-160 min, 5% to 35% buffer B; 160-170 min, 35% to 80% buffer B; 170-175 min, 80% buffer B; 176-180 min, 5% buffer B. The nanoliter liquid separation end is directly connected to the mass spectrometer. The peptides separated by liquid phase were sprayed into the nanoESI source and then entered a Q-Exactive HF tandem mass spectrometer for DDA and DIA analysis. DDA MS parameters were set to: (1) MS: 350–1,500 scan range (m/z); 60,000 resolution; 3e6 AGC target; 50 ms maximum injection time (MIT); 30 loop count; NCE 28; (2) HCD-MS/MS: 15,000 resolution; 1e5 AGC target; 100 ms MIT; charge exclusion, exclude 1, 7, 8, >8; filter dynamic exclusion duration 30 s; 2.0 m/z isolation window. The same nano-LC system and gradient are used for DIA analysis. DIA MS parameters were set to: (1) MS: 350–1,500 scan range (m/z); 20 ppm MS tolerance; 120,000 resolution; 3e6 AGC target; 50 ms MIT; 50 loop count; (2) HCD-MS/MS: 1.7 m/z isolation window; 30,000 resolution; 1e5 AGC target; automatic MIT; 50 loop count; filter dynamic exclusion duration 30s; stepped NCE: 22.5, 25, 27.5.

### Data analysis

DDA data were identified using MaxQuant (version 1.5.3.30) [123]. Peptide/protein entries that satisfied FDR ≤ 1% were used to build the final spectral library. Data was reviewed according to the UniProtKB/Swiss-Prot H. sapiens proteome database. Parameters were selected as follows: (i) Enzyme: Trypsin; (ii) Minimal peptide length: 7; (iii) PSM-level FDR and Protein FDR: 0.01; (iv) Fixed modifications: Carbamidomethyl (C); (v) Variable modifications: Oxidation (M); Acetyl (Protein N-term). DIA data was analyzed using Spectronaut [124], which uses iRT peptides to calibrate retention time. FDR was estimated using the mProphet scoring algorithm, which accurately reflects the matching degree of ion pairs. Then, based on the Target-decoy model applicable to SWATH-MS, the false positive control is completed with FDR not exceeding 1%. MSstats [125] screened DEPs according to a difference multiple ≥ 1.5 and P < 0.05 as the criteria for determining significant difference. The mass spectrometry proteomics data have been deposited to the ProteomeXchange Consortium via the PRIDE partner repository with the dataset identifier PXD018430.

## Supplementary Material

Supplementary Figure 1

Supplementary Table 1

Supplementary Table 2

Supplementary Table 3

Supplementary Table 4

Supplementary Table 5

Supplementary Table 6

Supplementary Table 7

Supplementary Table 8
