# Supplementary material for: Quantitative proteomics analysis of young and elderly skin with DIA mass spectrometry reveals new skin aging-related proteins
Source: Aging (Albany NY). 2020 Jun 29;12(13):13529–54. doi: 10.18632/aging.103461 (PMC7377841; doi:10.18632/aging.103461)
Supplement: Supplementary Figure 1 [file aging-12-103461-s007..pdf]

SUPPLEMENTARY FIGURE

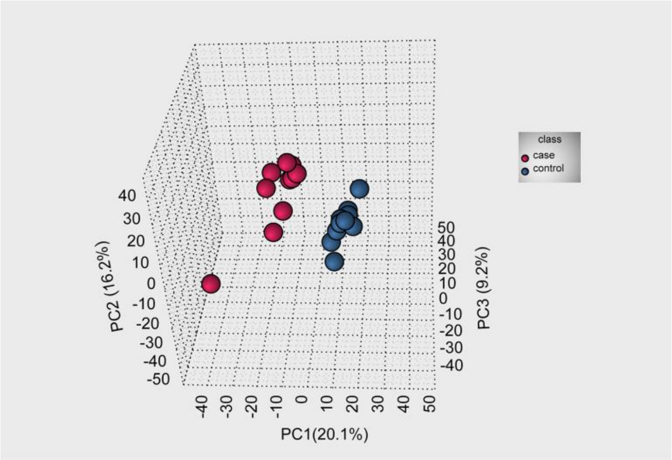

Supplementary Figure 1. Principal component analysis. The data revealed two separate DEP clusters that can distinguish the young from the elderly group.
